# Mosquito salivary gland protein preservation in the field for immunological and biochemical analysis

**DOI:** 10.1186/1756-3305-4-33

**Published:** 2011-03-08

**Authors:** A Fontaine, A Pascual, I Diouf, N Bakkali, S Bourdon, T Fusai, C Rogier, L Almeras

**Affiliations:** 1 Unité de recherche en biologie et épidémiologie parasitaires (URBEP) - UMR6236 - IFR48, Antenne Marseille de l'Institut de Recherche Biomédicale des Armées (IRBA), Le Pharo, BP 60109, 13 262 Marseille Cedex 07, France

## Abstract

Mosquito salivary proteins are involved in several biological processes that facilitate their blood feeding and have also been reported to elicit an IgG response in vertebrates. A growing number of studies have focused on this immunological response for its potential use as a biological marker of exposure to arthropod bites. As mosquito saliva collection is extremely laborious and inefficient, most research groups prefer to work on mosquito salivary glands (SGs). Thus, SG protein integrity is a critical factor in obtaining meaningful data from immunological and biochemical analysis. Current methodologies rely on an immediate freezing of SGs after their collection. However, the maintenance of samples in a frozen environment can be hard to achieve in field conditions. In this study, SG proteins from two mosquito species (*Aedes aegypti *and *Anopheles gambiae s.s*.) stored in different media for 5 days at either +4°C or room temperature (RT) were evaluated at the quantitative (*i.e*., ELISA) and qualitative (*i.e*., SDS-PAGE and immunoblotting) levels. Our results indicated that PBS medium supplemented with an anti-protease cocktail seems to be the best buffer to preserve SG antigens for 5 days at +4°C for ELISA analysis. Conversely, cell-lysis buffer (Urea-Thiourea-CHAPS-Tris) was best at preventing protein degradation both at +4°C and RT for further qualitative analysis. These convenient storage methods provide an alternative to freezing and are expected to be applicable to other biological samples collected in the field.

## Findings

Mosquitoes are responsible for a wide range of important diseases that cause morbidity and mortality in tropical and temperate regions [1,2]. Pathogen transmission occurs during the blood-feeding of infected mosquitoes, concomitant with salivary protein release [3]. Analysis of salivary mosquito contents using transcriptomic and proteomic tools [4-6] have revealed a panel of salivary molecules with anti-hemostatic and immuno-modulatory properties which facilitate blood meals by counteracting host's defences [6,7]. It was repeatedly demonstrated that mosquito salivary proteins could also elicit a host IgG response in natural conditions [8-10]. Thus, the potential use of these antigenic proteins as epidemiological markers for evaluating individual human exposure level to specific mosquito species is a major research area. Additionally, the identification of such vector-borne immunogenic proteins can lead to a panel of promising applications such as the evaluation of anti-mosquito strategies effectiveness, the mapping of new infestation areas, the estimation of disease transmission risk or the development of vaccines protecting the host against the transmission and establishment of pathogens [11,12]. As our aim is to identify biological markers of individual exposure to arthropod bites using correspondent antigenic materials, it was necessary to develop a convenient protocol to collect and preserve biological samples in the field.

The most common method used to obtain salivary proteins is salivary gland (SG) dissection [13-15]. Mosquito SGs contain a cocktail of enzymes and active proteins necessary for their blood-feeding that could alter salivary protein integrity [3,16]. To avoid protein degradation, SGs are generally collected on ice and stored at or below -20°C until needed [15,17]. However, maintaining samples in a frozen environment can be hard to achieve in field conditions.

Although hundreds of mosquito species have been reared in laboratories, relatively few have been continuously maintained through several generations in a caged environment [18]. Furthermore, continuous mass rearing is a tremendous task that requires particular skills and time and is also subject to biosafety considerations. Thus, to avoid the risk of mosquito settlement by bringing larvae outside their natural habitat in an area exempt of this species, collection of SG in the field appears more reasonable. However, SG dissection currently necessitates a large number of living mosquitoes *in situ *in close proximity to a freezer system to prevent protein degradation. Yet, in inter-tropical areas, mainly in sub-Saharan regions, some villages are highly isolated and frozen apparatus are not always available. Additionally, transport of SGE until a freezer system could take several hours and a continuous sample freezing could be uncertain. Therefore, alternative and convenient procedures need to be developed to preserve biological materials when a continuously cold environment would be hard to maintain. To this end, the preservation of SG proteins from two mosquito species (*Anopheles gambiae s.s *and *Aedes aegypti*) in different storage mediums and temperature conditions over 5 days have been evaluated quantitatively (*i.e*., ELISA) and qualitatively (*i.e*., SDS-PAGE and immunoblotting).

SGs from non-blood fed, 5-8 day-old adult *An. gambiae s.s*. (Kisumu strain [19]) and *Ae. aegypti *(Bora-Bora strain [20]) female mosquitoes bred in a laboratory under standard conditions (*i.e.*, 26°C and 60% humidity) at the "Institut de Recherche pour le Développement" (IRD, Montpellier)were dissected under a stereomicroscope, as previously described [15]. For each species, a total of 30 pairs of SGs were pooled and stored in each buffer and temperature condition. Briefly, samples were collected and stored 5 days at + 4°C or RT (about 21°C) either in a Phosphate Buffered Saline buffer supplemented with an anti-protease cocktail (one tablet of Complete, EDTA-free protease inhibitor cocktail (Roche Diagnostics, Indianapolis, USA) in 5 ml of PBS, PBSpi buffer), or in a cell lysis buffer (8 M urea (Sigma), 2 M thiourea (Sigma), 4% (w/v) CHAPS (Sigma) and 30 mM Tris (Sigma) adjusted to pH 8.5). After 5 days storage at + 4°C or RT, all the samples were preserved at -20°C until needed. As reference, 30 pairs of SGs from each species were collected in two independent replicates and placed on ice in PBS followed by freezing at -20°C [15].

Before testing the protein preservation conditions, the quantity of SG proteins collected was estimated for each sample. As protein degradation could occur in the different conditions tested, 2 pools of 30 pairs of SG for each mosquito species were collected in 2 independent experiments and conserved in reference conditions on ice in PBS. These pooled samples were then used for protein concentration measurements by the Lowry method (DC Protein assay Kit, Bio-Rad) according to the manufacturer's instructions. Protein concentration of *An. gambiae s.s. *and *Ae. Aegypti *samples were estimated at an average of 18.1 ± 2.0 μg (mean ± standard deviation) and 25.0 ± 2.4 μg per tube of 30 SG pairs, respectively.

SGs preserved in each condition were then disrupted by ultrasonication for 5 min on ice. Each sample was split into two equal quantities and precipitated with cold acetone (Sigma). One protein sample was suspended in bicarbonate buffer 0.1 M (pH 9.6) at 2 μg/mL, suitable for ELISA procedure, and the other was suspended in cell lysis buffer at 2.5 μg/μL, suitable for biochemical analysis [15]. To avoid several freeze-thaw cycles of the SGE samples and sera, ELISA and immunoblot experiments were run in parallel. Sera from 5 individuals (3 Senegalese and 2 Gabonese) regularly exposed to *An. gambiae *and *Ae. aegypti *mosquito bites and sera from 2 non-exposed French individuals who had not travelled abroad for the past 5 years were selected for this study. The protocol was approved by the ethical committee of Marseille (France) and by the Senegal National Ethics Committee (Dakar, Senegal). The informed consent of each participant was obtained at the beginning of the study, after a thorough explanation of its purpose.

ELISA analyses were performed as previously described [21]. Each serum (diluted at 1/50) was tested in duplicate and in control wells without SG extracts. IgG antibody levels are reported as adjusted OD (aOD), calculated for each serum as a mean OD value with SG extracts minus the OD value of the control wells.

**Figure 1:**
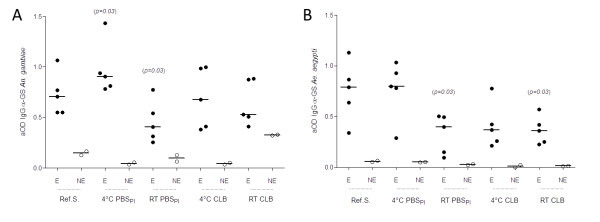
**Scatter plot graphs of human IgG responses from exposed (E) and non-exposed (NE) individuals against *An. gambiae *(A) and *Ae. aegypti *(B) salivary gland (SG) protein extracts according to different preservation conditions by Enzyme-Linked ImmunoSorbent Assay (ELISA)**. Antibody responses are represented by aOD: mean OD value of wells with salivary antigen minus mean OD value of wells without salivary antigen. Each point shows the aOD value for a single individual. Horizontal bars show medians. Differences between the reference sample (salivary glands collected on ice and store fresh at -20°C) and other preservation conditions were tested using Wilcoxon signed-rank test. *p-*values are indicated only when significant differences were observed. Ref. S: reference sample; CBL: cell lysis buffer.

A high IgG antibody response against *An. gambiae *SGs stored in the reference condition was observed by ELISA for exposed individuals (mean aOD ± standard deviation: 0.73 ± 0.21), in contrast to non-exposed individuals (0.15 ± 0.03). Comparable IgG antibody responses were obtained against *Ae. aegypti *SGs stored in the reference condition (0.75 ± 0.29 and 0.06 ± 0.01 for exposed and non-exposed individuals respectively; Figure 1A). These positive sera were considered suitable for the evaluation of the SGs' antigenicity.

The aOD of IgG antibody against SGs of both mosquito species from exposed individuals differed according to storage conditions. For *An. gambiae *samples, the highest mean aOD from exposed individuals was observed for SGs collected in PBSpi (0.97 ± 0.26), and to a lesser extent in cell lysis buffer (0.69 ± 0.30), when maintained for 5 days at +4°C. However, for samples collected in these same buffers (*i.e., *PBSpi or cell lysis buffer) but stored at RT, a decrease in aOD was observed (0.46 ± 0.21 and 0.64 ± 0.22 for samples collected in PBSpi and cell lysis buffer respectively; Figure 1A). For *Ae. aegypti *samples, only SGs collected in PBSpi and stored at +4°C showed an aOD from exposed individuals (0.77 ± 0.29) similar to that detected under the reference preservation condition (0.75 ± 0.29; Figure 1B). The aOD corresponding to IgG response against *An. gambiae *and *Ae. aegypti *SGs from non-exposed individuals was lower than the aOD detected with sera from exposed individuals. The aOD obtained with non-exposed sera against *An. gambiae *SGs preserved in cell lysis buffers at RT (0.33 ± 0.01) was unexpectedly higher than that obtained with *An. gambiae *SGs preserved in the other conditions. For *An. gambiae *SGs preservation conditions, significant increase and decrease (Wilcoxon signed rank test) of aOD from exposed individual sera were observed between reference sample and PBSpi at +4°C (*p = 0.03*) or PBSpi at RT (*p = 0.03*), respectively. For *Ae. aegypti *SGs preservation conditions, significant decreases (Wilcoxon signed rank test) of aOD from exposed individual sera were observed between reference sample and PBSpi at RT (*p = 0.03*) or cell lysis buffer at RT (*p = 0.03*) (Figure 1). Collectively, these results indicated that PBSpi at +4°C appeared to be the most efficient medium for preserving SG antigenicity during a 5-day storage period for further analysis by ELISA.

SG preservation was further evaluated by biochemical analysis including comparison of protein profiles by SDS-PAGE and immune response by immunoblots. For each preservation condition, 10 μg of SG protein was minimally labeled with CyDye as previously described [15,22] and separated by 12% SDS-PAGE (BioRad, Hercules, USA). Protein profiles were then analyzed using the ImageQuant™ TL software (GE Healthcare, UK), as previously described [23].

**Figure 2:**
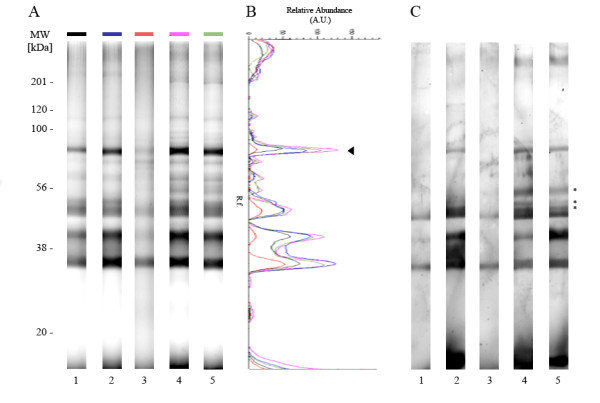
**Comparison of *An. gambiae* salivary gland profiles at the protein and antigenic level according to different preservation conditions.** (A) Comparative *An. gambiae *salivary gland (SG) protein profiles between different preservation conditions. Salivary gland proteins were separated on 12% SDS-PAGE gels. Each protein profile corresponds to a distinct preservation condition. Lane 1: SG dissected on ice and stored at -20°C in PBS (reference); lane 2: SG dissected at RT and stored 5 days at 4°C in PBS containing protease inhibitor cocktail (PBSpi); lane 3: SG dissected at RT and stored 5 days at RT in PBSpi; lane 4: SG dissected at RT and stored 5 days at 4°C in cell lysis buffer; lane 5: SG dissected at RT and stored 5 days at RT in cell lysis buffer. Standard molecular weights (MW) are indicated at the left side in kilodaltons (kDa). (B) Schematic representations of densitometric protein profiles from the 5 salivary gland preservation conditions. The line color corresponds to the colored box used at the top of each protein profile. The arrow head indicates the band that was used for abundance comparison. A.U.: Arbitrary Unit. R.f.: Relative front of migration. (C) IgG immune profiles against *An. gambiae *salivary gland proteins using the pooled sera from exposed individuals. The immunoblots were performed by transferring the SDS-PAGE gel shown in (A) onto a nitrocellulose membrane. Antigenic bands detected only in samples preserved in cell lysis buffer are indicated with an asterisk (*).

For *An. gambiae, *the diversity of protein bands, compared to the frozen reference, was independent of the preservation conditions used (Figure 2A), but large band intensity variations were observed dependent on the preservation conditions. For the same band, protein abundance between PBSpi RT and cell lysis buffer at 4°C could vary up to 11-fold (Figure 2B). Protein profiles with higher band diversity and intensity were obtained for samples preserved in cell lysis buffer either at +4°C or RT.

**Figure 3:**
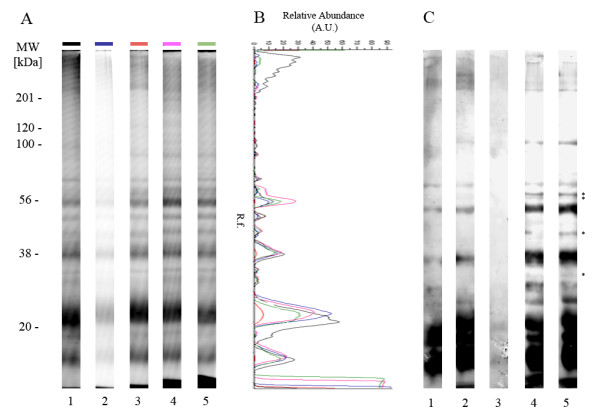
**Comparison of *Ae. aegypti* salivary gland profiles at the protein and antigenic level according to different preservation conditions.** (A) Comparative *Ae. aegypti *salivary gland (SG) protein profiles between different preservation conditions. Salivary gland proteins were separated on 12% SDS-PAGE gels. Each protein profile corresponds to a distinct preservation condition. Lane 1: SG dissected on ice and stored at -20°C in PBS (reference); lane 2: SG dissected at RT and stored 5 days at 4°C in PBS containing a protease inhibitor cocktail (PBSpi); lane 3: SG dissected at RT and stored 5 days at RT in PBSpi; lane 4: SG dissected at RT and stored 5 days at 4°C in cell lysis buffer; lane 5: SG dissected at RT and stored 5 days at RT in cell lysis buffer. Standard molecular weights (MW) are indicated at the left side in kilodaltons (kDa). (B) Schematic representations of densitometric protein profiles from the 5 salivary gland preservation conditions. The line color corresponds to the colored box used at the top of each protein profile. A.U.: Arbitrary Unit. R.f.: Relative front of migration. (C) IgG immune profiles against *Ae. aegypti *salivary gland proteins using the pooled sera from exposed individuals. The immunoblots were performed by transferring the SDS-PAGE gel shown in (A) onto a nitrocellulose membrane. Antigenic bands detected only in samples preserved in cell lysis buffer are indicated with an asterisk (*).

To further assess the consequences of sample preservation on the antigenic repertory, gels were transferred onto a nitrocellulose membrane (GE Healthcare) by semidry blotting [24] and further incubated with human pool sera from exposed individuals (n = 5, diluted at 1/100) and revealed mouse anti-human Fcg/IgG horseradish peroxidase (HRP) conjugated antibody (1/5 000, Beckman Coulter, USA) using an ECL Plus detection system (GE Healthcare). In accordance with SDS-PAGE analysis, immunoblots indicated that the antigenic repertoire appears better preserved in cell lysis buffer both at + 4°C or RT. In fact, numerous antigenic bands were detected only under this last preservation condition (Figure 2C). The intensity and diversity of antigenic profiles were most intense after cell lysis preservation when compared to the other conditions. Similar results were obtained for *Ae. aegypti *SG samples (Figure 3).

Surprisingly, in the reference condition, the low quality of protein and immune profiles suggested that protein degradation could occur in samples left at 4°C during SG collection periods, but the addition of a protease inhibitor cocktail could counteract this deleterious phenomenon. Interestingly, in *An. gambiae *samples, also the band at 70 kDa, reported as a major antigen [25], was detected of in all preservation conditions on the SDS-PAGE, this band was not recognized by the pool sera in the reference and PBSpi at RT conditions. The non-detection of this band could be reasonably attributed to an under detection limit of the immunoblot by this pool sera. Effectively, the band at 70 kDa is largely less abundant in the reference and PBSpi at RT conditions compared to three other conditions (PBSpi +4°C, cell lysis buffer +4°C or RT) accordingly to their corresponding densitometric protein profiles.

Although cell lysis buffer seems to be the best condition to preserve protein integrity for biochemical analysis at both + 4°C and RT, the lower aOD detected by ELISA from samples in these conditions could be attributed to the reagents from the cell lysis buffer (despite the acetone precipitation) interfering with the ELISA reaction, rather than a degradation of protein antigenicity. Effectively, Godfrin and collaborators demonstrate that high concentrations of CHAPS, Urea and Thiourea inhibit antigen binding to microplate surface and could also disturb antigen recognition by the specific antibodies in ELISA [26]. In addition, the combination of detergent and chaotropic agents in the cell lysis buffer induces protein denaturation leading to the loss of conformational epitopes [27]. The better conservation of these conformational epitopes in PBS is a supplementary argument to explain differences observed between preservation conditions in ELISA. Conversely, in immunoblots, epitopes recognized are mainly sequential due to reduction and denaturation of proteins, which could explain the disparate results obtained between the ELISA reactions and the SDS-PAGE or immunoblots.

To summarize, the cell lysis buffer solution seems to prevent protein degradation and preserve antigenicity at +4°C and also at RT. Nevertheless, despite cleaning the samples by acetone treatment, traces of this buffer could disrupt the ELISA experiments. In this specific case, protein preservation under PBSpi appeared to be more efficient to preserve SG antigenic proteins after 5 days of storage at +4°C. These convenient storage methods provide an alternative to freezing, which is hard to achieve under field conditions, and are expected to be applicable to biological samples in many systems.

## Abbreviations

IgG: Immunoglobulin G; RT: Room temperature; PBS: Phosphate Buffered Saline; PBSpi: Phosphate Buffered Saline supplemented with a protease inhibitor cocktail; EDTA: ethylenediaminetetraacetic acid; ELISA: Enzyme-Linked ImmunoSorbent Assay; HRP: Horseradish peroxidase; SDS-PAGE: Sodium dodecyl sulfate polyacrylamide gel electrophoresis.

## Competing interests

The authors declare that they have no competing interests.

## Authors' contributions

Conceived and designed the experiments: FA, AL, FT and RC. Performed the experiments: FA, PA and BS. Analyzed the data: FA, PA, AL and RC. Contributed reagents/materials/analysis tools: BS, DI and BN. Wrote the paper: FA, AL and RC. All authors read and approved the final version of the manuscript
